# Chemokine-like receptor 1 plays a critical role in modulating the regenerative and contractile properties of muscle tissue

**DOI:** 10.3389/fphys.2022.1044488

**Published:** 2022-11-17

**Authors:** Julian Boesch, Eliane Pierrel, Christian Lambert, Arno Doelemeyer, Julie Kreider, Nathalie Accart, Serge Summermatter

**Affiliations:** Musculoskeletal Diseases, Novartis Institutes for Biomedical Research, Novartis Pharma AG, Basel, Switzerland

**Keywords:** skeletal muscle, chemerin, strength, endurance, satellite cells

## Abstract

Musculoskeletal diseases are a leading contributor to mobility disability worldwide. Since the majority of patients with musculoskeletal diseases present with associated muscle weakness, treatment approaches typically comprise an element of resistance training to restore physical strength. The health-promoting effects of resistance exercise are mediated *via* complex, multifarious mechanisms including modulation of systemic and local inflammation. Here we investigated whether targeted inhibition of the chemerin pathway, which largely controls inflammatory processes *via* chemokine-like receptor 1 (CMKLR1), can improve skeletal muscle function. Using genetically modified mice, we demonstrate that blockade of CMKLR1 transiently increases maximal strength during growth, but lastingly decreases strength endurance. In-depth analyses of the underlying long-term adaptations revealed microscopic alterations in the number of Pax7-positive satellite cells, as well as molecular changes in genes governing myogenesis and calcium handling. Taken together, these data provide evidence of a critical role for CMKLR1 in regulating skeletal muscle function by modulating the regenerative and contractile properties of muscle tissue. CMKLR1 antagonists are increasingly viewed as therapeutic modalities for a variety of diseases (e.g., psoriasis, metabolic disorders, and multiple sclerosis). Our findings thus have implications for the development of novel drug substances that aim at targeting the chemerin pathway for musculoskeletal or other diseases.

## Introduction

Musculoskeletal diseases are a leading cause of years lived with disability globally (Sebbag et al., 2019; Cieza et al., 2021). Muscle weakness is a common manifestation in patients with musculoskeletal diseases, and there is increasing evidence that particularly lower skeletal muscle strength is a prognostic indicator for later disability (Newitt et al., 2016; Peterson et al., 2021). Intriguingly, a close relationship between low muscle strength and chronic inflammation has repeatedly been reported (Londhe and Guttridge, 2015; Tuttle et al., 2020; So et al., 2021). Moreover, inflammation inhibits protein synthesis and promotes catabolic processes in skeletal muscle, thereby actively driving the development of muscle atrophy and weakness (Costamagna et al., 2015; Draganidis et al., 2021).

Inflammatory responses are generally brought about *via* a complex interplay of various actors, but elevated levels of chemerin appear to be a common denominator in many settings with persistent low-grade inflammation (Rourke et al., 2013; Zhou et al., 2019). For instance, bioactive chemerin is detected in serum and tissue fluids of patients suffering from diseases with musculoskeletal manifestations such as rheumatoid arthritis or osteoarthritis (Zhao et al., 2018; Carrión et al., 2019; Cajas Santana et al., 2021; Gonzalez-Ponce et al., 2021). In these patients, chemerin induces the expression of additional inflammatory mediators such as IL-1β in chondrocytes, and TLR4 in synovial fluid, and stimulates leukocyte migration to the joint (Carrión et al., 2019).

Chemerin is a chemotactic protein predominantly expressed in adipose and liver tissue, and to a lesser extent, in skeletal muscle. It’s initially produced as a biologically inactive protein termed pre-prochemerin, which undergoes proteolytic cleavage to form prochemerin, and following further extracellular processing, is subsequently converted to the fully bioactive chemerin protein (Kennedy and Davenport, 2018). In humans, there are at least three active products, which have been detected in biological fluids: chemerin156, chemerin157, and chemerin158 (Fischer and Beck-Sickinger, 2022). Further proteolytic events can cleave active chemerin to inactive, or low activity proteins (Fischer and Beck-Sickinger, 2022). Bioactive chemerin is the endogenous ligand for two signaling receptors, chemokine-like receptor 1 (CMKLR1) and G protein-coupled receptor 1 (GPR1) (Kennedy and Davenport, 2018). There is a third chemerin receptor, C-C chemokine receptor-like 2 (CCRL2), which is thought to serve as a chemerin membrane anchoring protein that increases local chemerin concentrations and presents the ligand to CMKLR1 or GPR1 expressing cells rather than directly mediating chemerin signaling (Fischer and Beck-Sickinger, 2022). CMKLR1 acts as a main receptor for chemerin as it couples to G_i_ proteins, inhibits the synthesis of cyclic adenosine monophosphate, and leads to the recruitment of arrestin-3 with subsequent internalization of the receptor (De Henau et al., 2016). Further downstream, CMKLR1 activation leads to phosphorylation of ERK1/2, p38 MAPK, and Akt (De Henau et al., 2016). By contrast, GPR1 seems to be an unusual G protein-coupled receptor, and it remains unclear whether it induces G protein signaling (Fischer and Beck-Sickinger, 2022). Published data suggest that activated GPR1 rapidly recruits arrestin and is then internalized (De Henau et al., 2016). While the effects of chemerin have been studied on various cell types, specific data pertaining to the effect on skeletal muscle are scarce. The limited data available indicate that exposing skeletal muscle to elevated levels of chemerin induces mitochondrial autophagy and insulin resistance; suggesting chemerin exerts anti-anabolic and pro-catabolic effects on skeletal muscle (Sell et al., 2009; Becker et al., 2010; Xie et al., 2015).

To date, exercise remains the most efficacious intervention to treat musculoskeletal conditions and improve functional parameters (e.g., strength, gait speed, static and dynamic balance, and fall risk reduction) (Cup et al., 2007; Voet et al., 2013) (Papa et al., 2017). At the same time, exercise regimens have been shown to effectively decrease the levels of circulating chemerin (Stefanov et al., 2014; Ashtary-Larky et al., 2021). We thus hypothesized that targeted ablation of CMKLR1 might mimic specific beneficial effects of exercise and improve skeletal muscle function.

## Material and methods

### Animals

All animal studies described were performed according to the official regulations effective in the Canton of Basel-City, Switzerland. CMKLR1 knockout mice were backcrossed to C57BL/6 mice from Charles River Laboratories for >10 generations. C57BL/6 mice and CMKLR1 KO mice on the same C57BL/6 background were bred at Charles River Laboratories. Male animals were used for all studies. 8, 12 and over 15 weeks old mice were denoted as “young”, “adult” and “full-grown”, respectively. All animals were acclimatized to the research facility in Basel (Switzerland) for 7 days. They were housed at 25 °C with a 12:12 h light-dark cycle and were fed a standard laboratory diet containing 18.2% protein and 3.0% fat with an energy content of 15.8 MJ/kg (Nafag, product # 3890, Kliba, Basel, Switzerland). Food and water were provided *ad libitum*.

For studies with the CMKLR1 antagonist 2-(anaphthoyl) ethyltrimethylammonium iodide (α-NETA), full-grown C57BL/6 mice were subcutaneously injected with 10 mg/kg α-NETA formulated in 10% sulfobutyl ether β-cyclodextrin. Muscle samples were harvested 24 h after the injection.

### Isometric muscle force

Evoked isometric force was measured non-invasively *via* electrical stimulation of the hind leg through transcutaneous electrodes as previously described (Summermatter et al., 2017). In brief, the animals were anesthetized, the foot was placed on a homemade pedal connected to a force transducer, and the force generated in response to electrical stimulation through the skin was recorded. Various frequencies ranging from 10 to 120 Hz were sequentially applied, and tetanic forces recorded at each frequency.

### Inverted grip strength

Animals were placed on a horizontal rotatable metal grid approximately 30 cm over a cushion. The grid was flipped upside down, and the time taken until the animals fell onto the cushion recorded.

### Gait analyses

Gait parameters of freely moving mice were measured using the Catwalk gait analysis system (Noldus Information Technology, Netherlands) as described previously (Parvathy and Masocha, 2013). Briefly, each mouse was placed individually in the CatWalk walkway and allowed to freely walk along an illuminated walkway glass plate. Animal contact with the glass plate resulted in changes in light intensity, which was captured by a camera positioned under the walkway. Mice were habituated to the Catwalk walkway prior to the start of the study. Gait parameters at young and adult phase were measured. Three runs for each animal were recorded per time point for further analysis by the software (CatWalk XT 10.6, Noldus Information Technology, Netherlands). The CatWalk calculates different parameters based on footprints, or on time recorded.

### Gene expression profiling

Total RNA was extracted from skeletal muscle using TRIzol reagent (Invitrogen). Reverse transcription was performed with random hexamers on 1 μg of total RNA using a high-capacity reverse transcription kit (Applied Biosystems), and the reaction mixture was diluted 20-fold. RT-PCRs were performed in duplicates in 384-well plates on an AB7900HT cycler (Applied Biosystems) using specific TaqMan probes (Applied Biosystems). Data were normalized to two housekeeping genes using the ΔΔCT threshold cycle (CT) method. Fluorescence was measured at the end of each cycle, and after 40 reaction cycles, a profile of fluorescence versus cycle number was obtained. Automatic settings were used in most cases to determine the CT. The comparative method using 2^−ΔΔCT^ was applied to determine the relative expression. All results are expressed as fold changes over controls.

### Histology

Tibialis Anterior muscles were harvested and processed for paraffin embedding. For histology, muscles were cut into 10 μm cross-sections. The immunostaining was performed in the automate Ultra Discovery according to the manufacturer’s recommendations (Roche Diagnostics, Rotkreuz, Switzerland). Briefly, sections were de-waxed and antigen retrieval was obtained after incubation in the cell conditioner 1 (Roche) for 12 min at 95°C. After blocking in 6% normal goat serum and washing in the reaction buffer, slides were incubated with the primary antibody solution against Pax7 (ab187339, Abcam) for 1 h at room temperature to detect satellite cells. After a post-fixation in 0.05% glutaraldehyde for 8 min, antibody detection was performed using an anti-rabbit-HQ (Roche) incubated for 12 min, followed by an anti-HQ-HRP (Roche) incubated for 12 min. After several washes, the sections were counterstained with Hematoxylin II and Post Counterstained with Bluing reagents (Roche). After washing and dehydration steps samples were mounted with Pertex.

### Image analysis

Glass slides were scanned with a Philips Ultra-Fast Scanner (Philips AG, Horgen, Switzerland) at 400x magnification resulting in image pixel dimensions of 0.25 µm × 0.25 µm. For the quantitative assessment of Pax7 positive nuclei, the HALO image analysis platform (Indica Labs, Albuquerque, NM, United States) was used. The image analysis was performed in two steps: first detecting the regions of interest (ROIs) in the tissue (excluding areas that may result in artifacts, e.g., tissue folds) and subsequently detecting nuclei positive and negative for PAX7. For the ROI detection a machine learning based classifier (DenseNet, HALO AI) has been devised and trained to recognize three different classes: muscle tissue, exclusion areas and whitespace. In a second step, nuclei positive and negative for PAX7 have been detected in the previously defined region of muscle tissue using the HALO module Multiplex IHC v3.0.1.

### Statistical analyses

Statistical analyses were performed as indicated using GraphPad Prism version 7.03 (GraphPad Software, Inc., La Jolla, CA). Differences were considered significant when the probability value was <0.05.

## Results

### Blockade of CMKLR1 regulates maximal strength and strength endurance during growth

**FIGURE 1:**
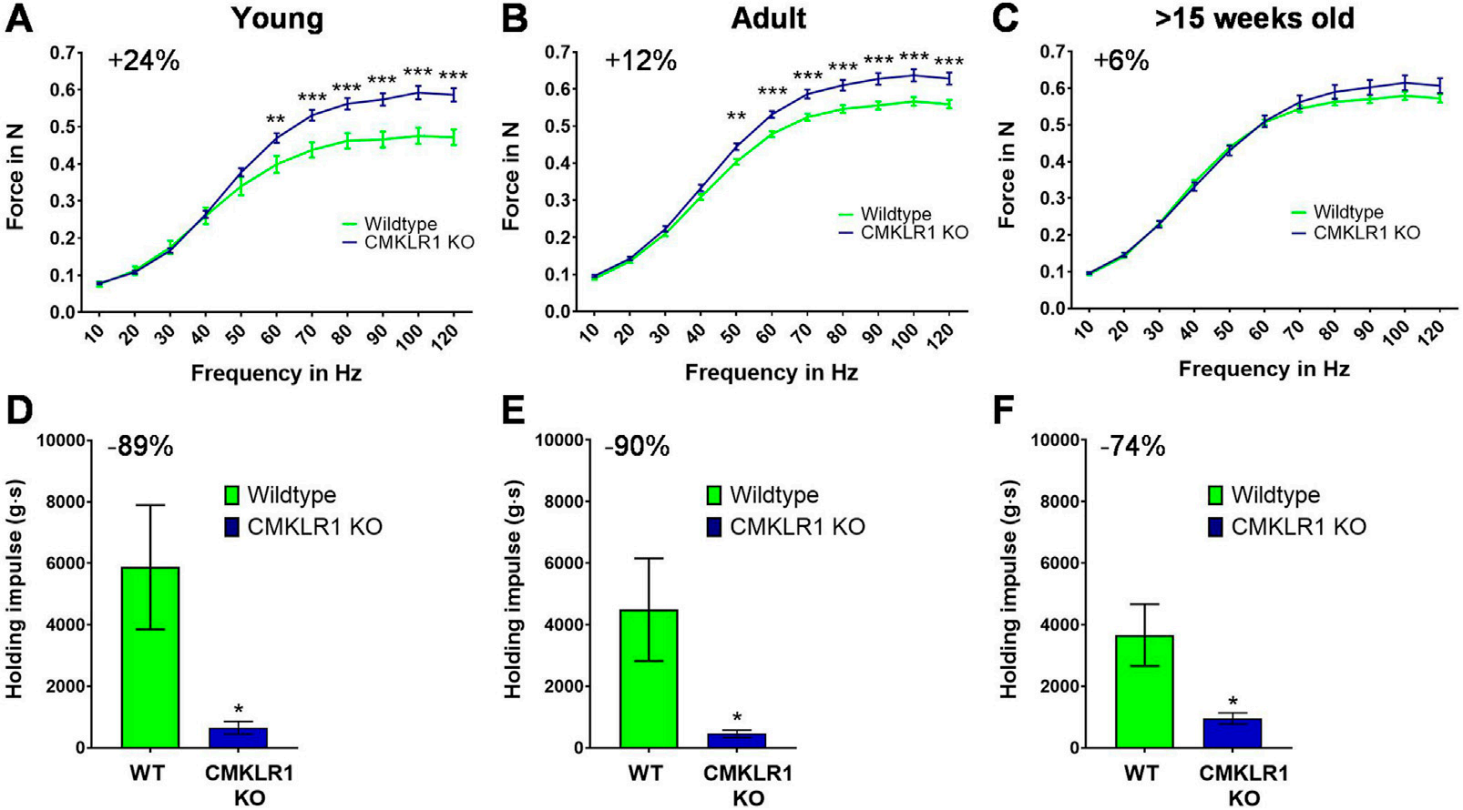
Blockade of CMKLR1 increases muscle strength at the expense of strength endurance Isometric muscle strength of the hind limb of young **(A)**, adult **(B)** and full grown **(C)** CMKLR1 knockout and control mice. Values are expressed as means ± SEM; **: *p* < 0.01, ***: *p* < 0.001 versus wild-type control mice by unpaired *t*-test; n = 17–21 per group. Vertical inverted grip strength in young **(D)**, adult **(E)** and full grown **(F)** CMKLR1 knockout and control mice. Values are expressed as means ± SEM; *: *p* < 0.05, versus wild-type control mice by unpaired *t*-test; n = 10 per group.

Chemerin is a secreted protein that induces mitochondrial autophagy and insulin resistance in skeletal muscle (Sell et al., 2009; Becker et al., 2010; Xie et al., 2015). Since CMKLR1 is the most expressed chemerin receptor in muscle (https://www.ebi.ac.uk/gxa/home), we evaluated whether blocking CMKLR1 has an impact on skeletal muscle function. We found that young (8-weeks-old) CMKLR1-deficient mice were able to generate approximately 24% higher isometric force than their aged-matched controls (Figure 1A). This adaptation was observed exclusively following electrical stimulation of the hind limb muscles at high frequencies, which reflects maximal strength. By contrast, no differences in force were detected at low frequency stimulations.

As the mice grew older, CMKLR1 knockout mice retained their elevated maximal strength, while control animals continuously caught up. In fact, the difference diminished by a factor of two during adulthood (12 weeks of age) (Figure 1B) and disappeared completely once the animals were full-grown (after 15 weeks of age) (Figure 1C). The observed disparity in maximal strength was not due to altered body weight, which was indistinguishable between CMKLR1 knockout and control mice at any age (Supplementary Table S1). These data indicate that blockade of the chemerin receptor CMKLR1 accelerates the gain in maximal isometric strength during physiological muscle growth.

We next evaluated whether the absence of CMKLR1 modulates other parameters relevant for physical activity. To this end, we assessed strength endurance by using an inverted grip strength test. Intriguingly, inhibition of chemerin signaling reduced the latency to when an animal fell by 89, 90 and 74% in young, adult, and full-grown mice, respectively (Figures 1D,E). Abrogating CMKLR1 signaling thus lastingly impairs strength endurance performance.

### Gait patterns of young CMKLR1 knockout mice resemble those of adult controls

**FIGURE 2:**
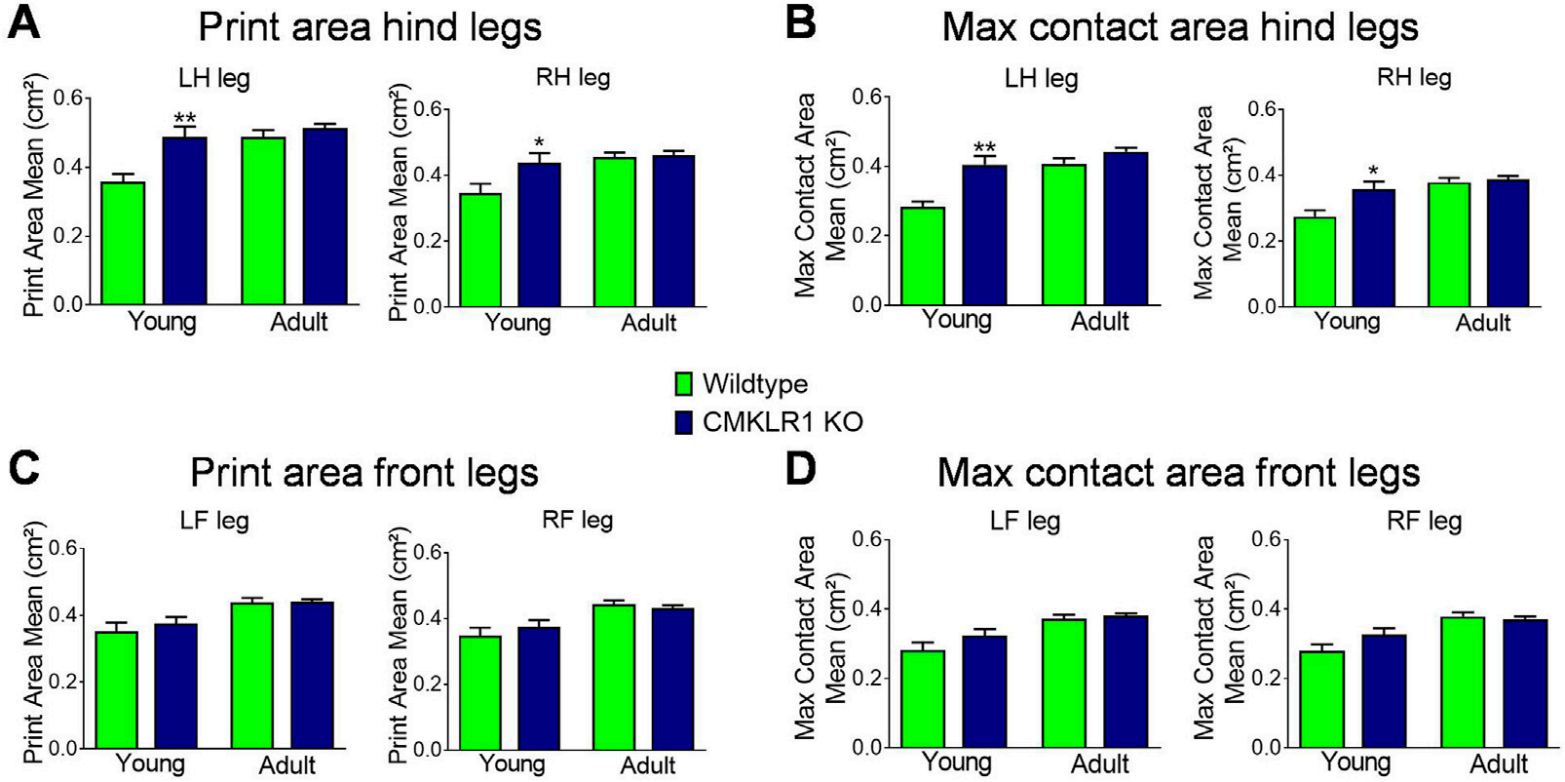
CMKLR1 inhibition alters mobility parameters Print area mean **(A)** and maximum contact area **(B)** of the hind legs of wild type and CmklR1 KO mice at young and adult stage. Print area mean **(C)** and maximum contact area **(D)** of the front legs of wild type and CmklR1 KO mice at young and adult stage Values are expressed as means ± SEM; *: *p* < 0.05, **: *p* < 0.01, versus wild type control mice by unpaired *t*-test; n = 7–12 per group.

The ability to generate and maintain force can significantly affect gait patterns. Since both strength parameters were altered concomitantly in young and adult mice, we assessed gait parameters in these two groups. In young animals lacking a functional chemerin receptor, the print area of the left and right hind legs was markedly larger compared to controls, reaching levels comparable to adult control and CMKLR1 knockout mice (Figure 2A). In line with the larger print area, the maximum contact area, which is the area of the foot touching the ground during the time of maximum contact, was also larger in young CMKLR1 knockout mice as compared to control animals (Figure 2B). We were unable to observe any significant differences between adult control and CMKLR1 knockout mice for any of the parameters tested. Furthermore, no significant differences were detected for the front legs (Figures 2C,D), or for any other gait-related key parameter (Supplementary Table S2). The observed changes in gait parameters demonstrate that young mice deficient in CMKLR1 adopt a walking pattern that resembles adult control mice.

### The number of satellite cells is reduced in skeletal muscle of CMKLR1 knockout mice

**FIGURE 3:**
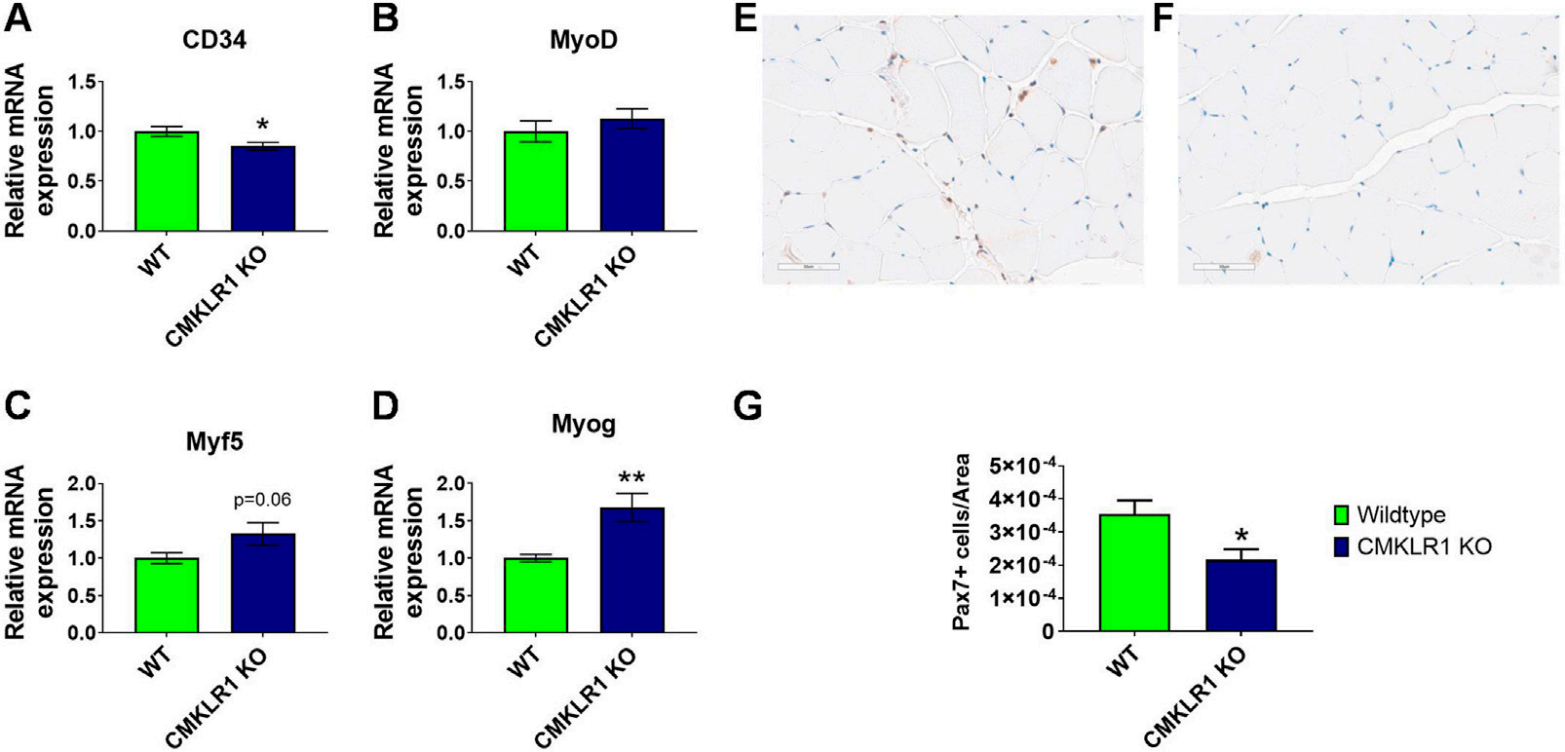
CMKLR1 inhibition modulates myogenesis Relative mRNA levels of myogenesis markers in mouse gastrocnemius complex of wild type and CmklR1 KO mice. CD34 as marker for quiescence **(A)**, MyoD **(B)** and Myf5 **(C)** as early-stage markers of myogenic differentiation, and myogenin **(D)** as a late-stage marker. Representative section with PAX7 staining of a control animal **(E)** and a CMKLR1 knockout mouse **(F)** with machine-learning-based segmentation and classification of PAX7 positive (brownish) and negative nuclei (blue). Quantification with group comparison **(G)**. Values are expressed as means ± SEM; *: *p* < 0.05, **: *p* < 0.01 versus wild type mice by unpaired *t*-test; n = 8–10 per group.

While the changes in maximal strength and gait occur specifically during the growth phase, the reduction in strength endurance extends beyond the growth phase. We therefore sought to understand this prolonged re-programming induced by blockade of CMKLR1. Satellite cells play a central role in controlling skeletal muscle physiology, and depletion of resident muscle stem cells negatively impacts the volume of physical activity (Englund et al., 2020). We thus performed molecular and histological analyses on skeletal muscle of full-grown animals to evaluate the contribution of satellite cells to the observed phenotype. Gastrocnemius muscle of mice deficient in CMKLR1 showed a pattern of myogenesis markers that was distinct from controls: significantly reduced CD34 (Figure 3A), unaltered MyoD and Myf5 (Figures 3B,C), and significantly increased myogenin mRNA levels (Figure 3D). Since CD34 is a marker of satellite cell quiescence, and its expression is reduced upon satellite cell activation (Beauchamp et al., 2000; Zammit et al., 2006), these data suggest that loss of CMKLR1 promotes the exit of satellite cells from quiescence. In accordance with this, early markers of myogenic commitment and differentiation such as Myf5 tended to be increased (Figure 3C), while myogenin as a late-stage marker for muscle differentiation (Zammit et al., 2006) was significantly increased (Figure 3D). This transcriptional profile pointed towards activation and subsequent myogenic commitment of satellite cells in the absence of an intact signaling *via* CMKLR1. Moreover, when we analyzed the number of Pax7 positive satellite cells with a machine-learning-based approach (Figures 3E,F), we found that CMKLR1 knockout mice displayed a significantly lower number of these cells (Figure 3G). Collectively, these data underpin that knockout of CMKLR1 drives satellite cells from quiescence into myogenesis.

### Loss of CMKLR1-mediated chemerin signaling promotes expression of genes governing calcium regulation in skeletal muscle

**FIGURE 4:**
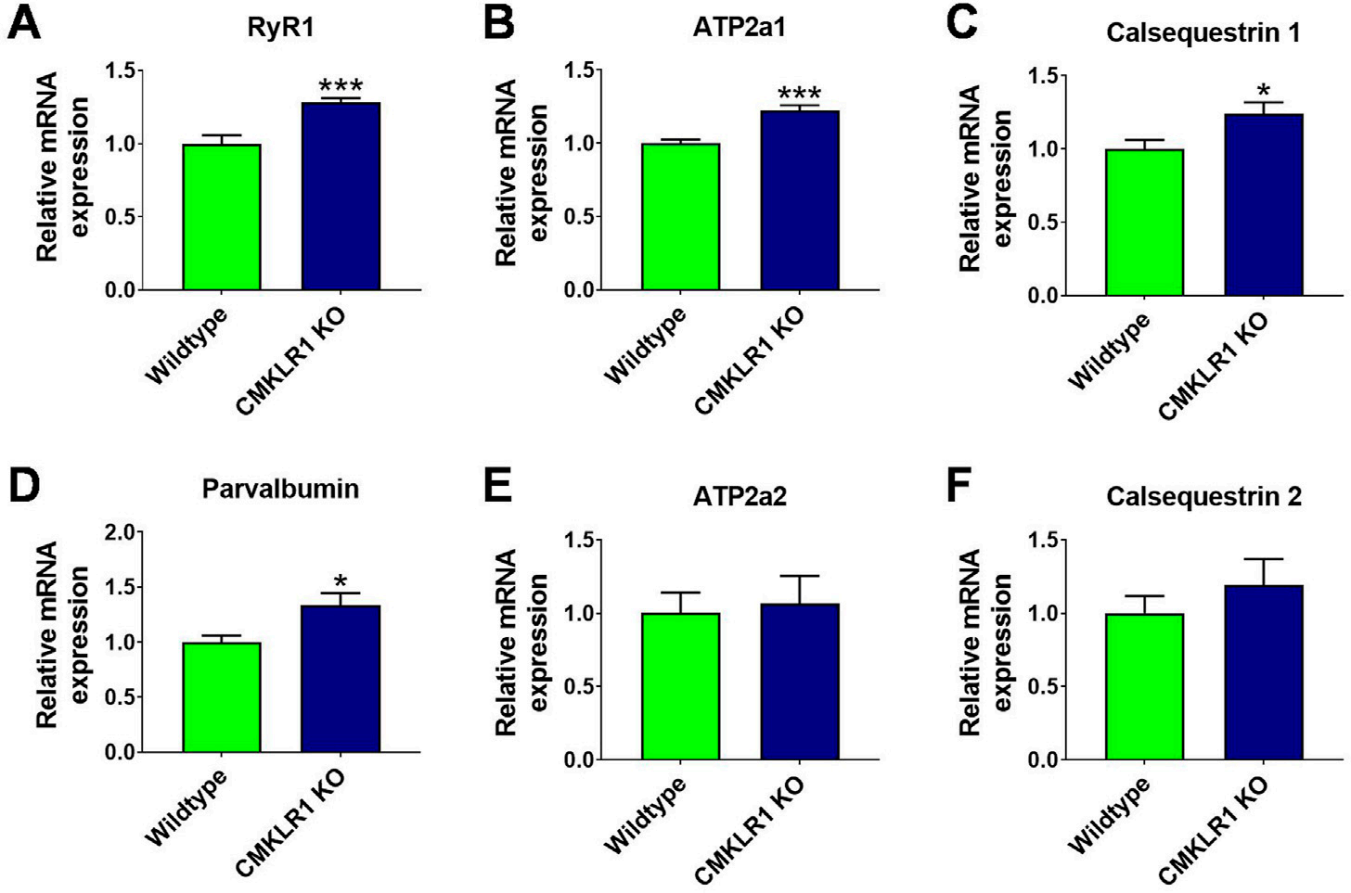
Abrogating signaling through CMKLR1 induces expression patterns characteristic of fast-twitch muscles Relative mRNA levels of genes governing rapid calcium release and uptake measured in mouse gastrocnemius complex of wild type and CmklR1 KO mice: Ryanodine receptor for calcium release **(A)**, SERCA1 for calcium reuptake **(B)**, calsequestrin 1 **(C)** and parvalbumin **(D)** for calcium storage and buffering. SERCA2 **(E)** and calsequestrin 2 **(F)** are the prevalent isoforms in fibers with slow calcium turnover. Values are expressed as means ± SEM; *: *p* < 0.05, **: *p* < 0.01, ***: *p* < 0.001 versus wild type mice by unpaired *t*-test; n = 9–10 per group.

To gain further insights into the adaptive molecular mechanisms that persist beyond the growth phase, we measured the mRNA expression of key components regulating muscle contraction. Inhibition of CMKLR1 markedly enhanced the expression of genes involved in calcium regulation in full-grown animals. In gastrocnemius muscle, blockade of CMKLR1 increased the mRNA expression of the ryanodine receptor, indicating a higher capacity to release calcium from the sarcoplasmic reticulum to initiate muscle contraction, and of SERCA1, which is responsible for subsequent calcium reuptake (Figures 4A,B). In addition, the expression of genes, such as calsequestrin 1 and parvalbumin, involved in fast muscle relaxation, which is characteristic of a resistance-trained, but fatigue-prone muscle, were significantly elevated in the absence of functional CMKLR1 (Figures 4C,D). In contrast, the other isoforms of these calcium-handling genes did not significantly differ between the two genotypes (Figures 4E,F). Hence, the targeted transcriptional profiling of skeletal muscle of CMKLR1 knockout mice further corroborates a role for CMKLR1 in regulating transcriptional adaptations that favor an accelerated development of maximal but hamper the development of strength endurance.

### Inhibition of CMKLR1 reduces inflammatory markers in skeletal muscle

As a final verification that abrogating CMKLR1 alters inflammation in our tissue of interest, we measured the gene expression of inflammatory markers in skeletal muscle. Blockade of CMKLR1 tended to decrease the muscle mRNA expression of TNFα, and significantly reduced the levels of CD68 and RELA (p65) (Supplementary Figures S1A–C). These data underscore an anti-inflammatory effect of CMKLR1 inhibition in skeletal muscle.

### Acute pharmacological inhibition of CMKLR1 has limited effects on markers of myogenesis

So far, no approved drugs to suppress CMKLR1 activity are available commercially. However, a small molecule with the structure 2-(anaphthoyl) ethyltrimethylammonium iodide (α-NETA) has been found to suppress CMKLR1 signaling in preclinical studies (Graham et al., 2014). We thus evaluated the effect of α-NETA on markers of myogenesis, calcium regulation and inflammation. Administration of α-NETA significantly decreased CD34 and MyoD expression in skeletal muscle (Supplementary Figures S2A,B) without altering any of the other markers (Supplementary Figures S2C–M).

## Discussion

Chronic inflammation, either local or systemic, is associated with lower skeletal muscle mass and strength (Tuttle et al., 2020), causally driving the development of muscle atrophy and weakness by inhibiting muscular protein synthesis, promoting catabolic processes and impairing regeneration (Degens, 2010) (Costamagna et al., 2015; Draganidis et al., 2021). Interestingly, many disorders with chronic low-grade inflammation such as cardiovascular disease, cancer, alimentary tract disorders, metabolic syndrome, rheumatoid arthritis and osteoarthritis feature elevated levels of the adipokine chemerin (Stojek, 2017; Zhao et al., 2018; Carrión et al., 2019; Treeck et al., 2019). In skeletal muscle, exposure to high levels of chemerin provokes metabolic perturbations (Sell et al., 2009; Becker et al., 2010; Xie et al., 2015). Consequently, secreted factors with pro-inflammatory actions like chemerin are considered promising therapeutic targets (Di Felice et al., 2020). We have now demonstrated that blockade of CMKLR1 modulates skeletal muscle function by favoring the development of maximal strength over strength endurance. Furthermore, we provided first insights into the molecular and histological adaptations that drive the development of this contractile phenotype. Although increasing maximal strength might be beneficial for patients with muscle weakness, reducing strength endurance constitutes a maladaptive trait. Thus, our findings have major implications for the development of CMKLR1 antagonists to treat the various diseases with aberrant circulating levels of chemerin.

A key finding of our study is that blockade of CMKLR1 remodels the contractile properties of skeletal muscle. Muscle fibers that can generate high force typically express high levels of ryanodine receptor 1 (RyR1) (Froemming et al., 2000). These receptors are huge ion channels that transport calcium from the sarcoplasmic reticulum into the cytosol. Consequently, more calcium can be released in response to motor neuron activation (Baylor and Hollingworth, 2003). Once released from the sarcoplasmic reticulum (SR), calcium binds to troponin, thereby re-directing tropomyosin, exposing the myosin-binding sites, and allowing muscle contraction. Moreover, muscle fibers generating high maximal force are endowed with a high amount of sarcoplasmic/endoplasmic reticulum calcium-ATPase 1 (SERCA1) that pumps calcium back into the SR, with calsequestrin1 that facilitates the re-uptake of calcium into the sarcoplasmic reticulum and with parvalbumin that sequesters calcium and enhances calcium re-uptake (Froemming et al., 2000). The interplay of these factors enables rapid muscle contraction as well as rapid relaxation. Our data show that knockout of CMKLR1 specifically promotes expression of factors that expedite efficient calcium release and reuptake. This molecular pattern is characteristic of resistance-trained muscles that can generate high peak forces but tend to fatigue rapidly. Consistently, mice lacking CMKLR1 exhibit decreased strength endurance and their gene signatures are diametrically opposed to genetic models that promote an endurance-trained phenotype. For instance, overexpression of PGC1a, which reproduces key features of endurance training, reduces the levels of Ryr1, SERCA1, parvalbumin, and calsequestrin 1 in skeletal muscle (Summermatter et al., 2012).

Our study additionally sheds light on the action of chemerin on skeletal muscle myogenesis. We demonstrate that knockout of CMKLR1 decreased the expression of the quiescence marker CD34 and increased the expression of myogenin, which is a marker of late-stage differentiation (Zammit et al., 2006). In line with our *in vivo* data, exposure of mouse C2C12 skeletal muscle cells to chemerin *in vitro* has been found to inhibit muscle specific transcription factors such as myogenin, while promoting the mRNA expressions and protein concentrations of adipogenic factors, thereby shifting the commitment of these cells away from myogenesis (Li et al., 2015). Another independent report showed that chemerin suppressed differentiation of C2C12 cells through extracellular-signal regulated kinase-1/2 (ERK1/2) and mammalian target of rapamycin (mTOR) signaling pathways and promoted proliferation (Yang et al., 2012). Surprisingly, decreasing CMKLR1 expression by adenoviral-delivered small-hairpin RNA (shRNA) impaired the differentiation of C2C12 myoblasts into mature myotubes and reduced the mRNA expression of myogenic regulatory factors such as myogenin and MyoD (Issa et al., 2012). In the same study, the authors used 12-week old CMKLR1 knockout mice and found unaltered levels of myogenin, but reduced levels of MyoD in gastrocnemius muscle (Issa et al., 2012). Therefore, to further clarify the role of the chemerin/CMKLR1 pathway in myogenesis, we have additionally measured the actual number of skeletal muscle precursor cells and found reduced levels of Pax7 positive satellite cells in CMKLR1 knockout mice. The reduced number of satellite cells in CMKLR1 knockout mice might contribute to the impairment in strength endurance as satellite cell depletion throughout adulthood has been reported to reduce endurance performance (Englund et al., 2020).

In the light of the emerging evidence that dysregulated chemerin levels are associated with various, non-communicable diseases (Stojek, 2017; Kennedy and Davenport, 2018; Treeck et al., 2019), the interest in pharmacological CMKLR1 inhibitors is growing (Imaizumi et al., 2019; Kumar et al., 2019). A small molecule with the chemical structure 2-(anaphthoyl) ethyltrimethylammonium iodide (α-NETA), shown to inhibit CMKLR1, can suppress CNS autoimmune inflammatory disease and has been suggested as a novel approach to prevent or treat multiple sclerosis; a disease that is associated with loss of muscle mass and function (Kennedy and Davenport, 2018; Kumar et al., 2019). Our data show a consistent reduction in CD34 expression in response to α-NETA and knockout of CMKLR1. Additionally, α-NETA reduced the levels of the early differentiation marker MyoD. A possible reason for this finding is that α-NETA inhibits aldehyde dehydrogenase more potently than CMKLR1 (Graham et al., 2014). Skeletal muscle progenitors with elevated expression of aldehyde dehydrogenase are endued with increased myogenic differentiation capacity when compared to cells with low expression (Vella et al., 2011). We thus conclude that compounds with higher selectivity and longer treatment duration will be needed to fully elucidate the impact of pharmacological CMKLR1 inhibition on skeletal muscle.

Overall, our findings unravel a critical role for CMKLR1 in skeletal muscle remodeling. Ablation of CMKLR1 promotes myogenesis, depletes the number of satellite cells, and shifts the contractile properties from an endurance to a resistance trained phenotype. While the increase in maximal strength is limited to the growth phase, the concomitant loss in strength endurance capacity persists for longer. Our results therefore reveal a novel feature of the chemerin pathway that extends beyond its established role in driving inflammation. However, these findings urge caution since in the long run, inhibition of CMKLR1 impairs strength endurance and thus, might promote muscle fatigue and reduce mobility in patients treated with CMKLR antagonists. Thus, our data highlight a novel avenue to remodel skeletal muscle function during muscle growth or regeneration, as well as a potential pitfall that is associated with the development of CMKLR1 antagonists for muscle and non-muscle diseases.

## Data Availability

The original contributions presented in the study are included in the article/Supplementary Material, further inquiries can be directed to the corresponding author.

## Supplementary material

SUPPLEMENTARY FIGURE S1 Knocking out CMKLR1 decreases markers of inflammation in skeletal muscle. Relative mRNA levels of inflammatory markers measured in mouse gastrocnemius complex of wild type and CmklR1 KO mice: TNFα (A), CD68 (B) and RELA (C). Values are expressed as means ± SEM; *: *P* < 0.05 versus wild type mice by unpaired t-test; n= 9-10 per group.

SUPPLEMENTARY FIGURE S2 Acute pharmacological inhibition of CMKLR1 has limited effects on markers of myogenesis, calcium handling and inflammation. Relative mRNA levels of genes involved in myogenesis, calcium regulation and inflammation measured in mouse gastrocnemius complex of mice treated with vehicle or 10 mg/kg body weight α-NETA: Markers of myogenesis (A-D), calcium regulation (E-J) and inflammation (K-M). Values are expressed as means ± SEM; ***: *P* < 0.001 versus vehicle-treated mice by unpaired t-test; n= 8 per group.

SUPPLEMENTARY TABLE S1 Body weight.

SUPPLEMENTARY TABLE S2 Gait parameters investigated.
