# Supplementary figures and images for: Chemokine-like receptor 1 plays a critical role in modulating the regenerative and contractile properties of muscle tissue
Source: Front Physiol. 2022 Nov 17;13:1044488. doi: 10.3389/fphys.2022.1044488 (PMC9713634; doi:10.3389/fphys.2022.1044488)

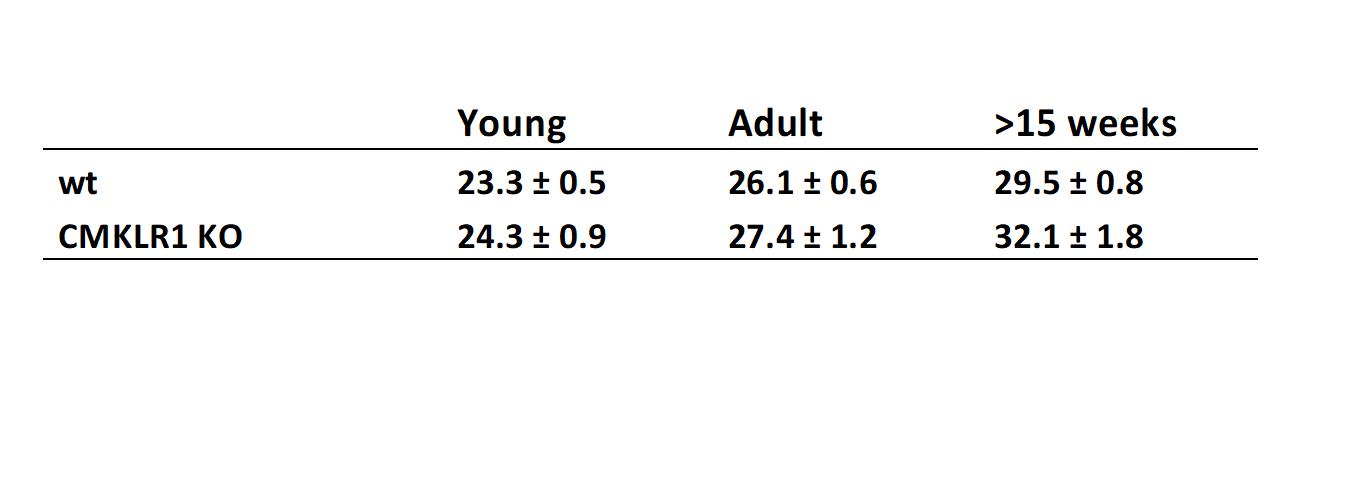

Supplement: Supplementary file 1 [file Image3.jpg]

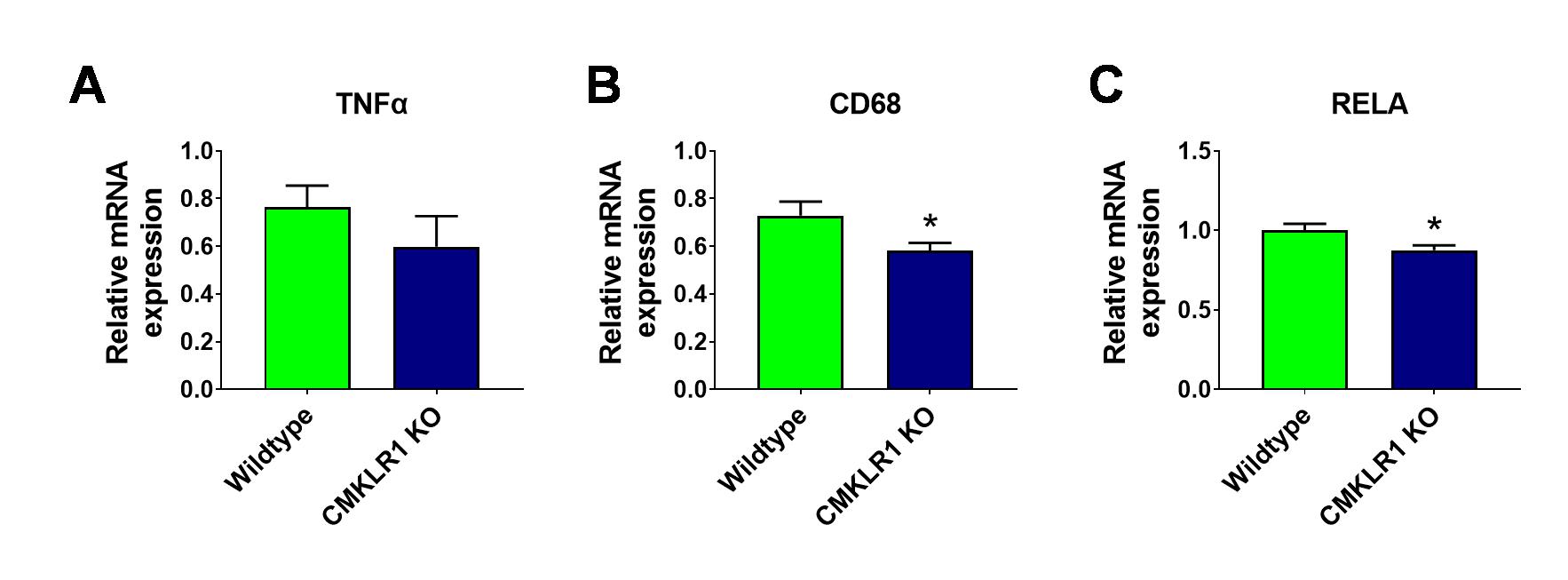

Supplement: Supplementary file 2 [file Image1.JPEG]

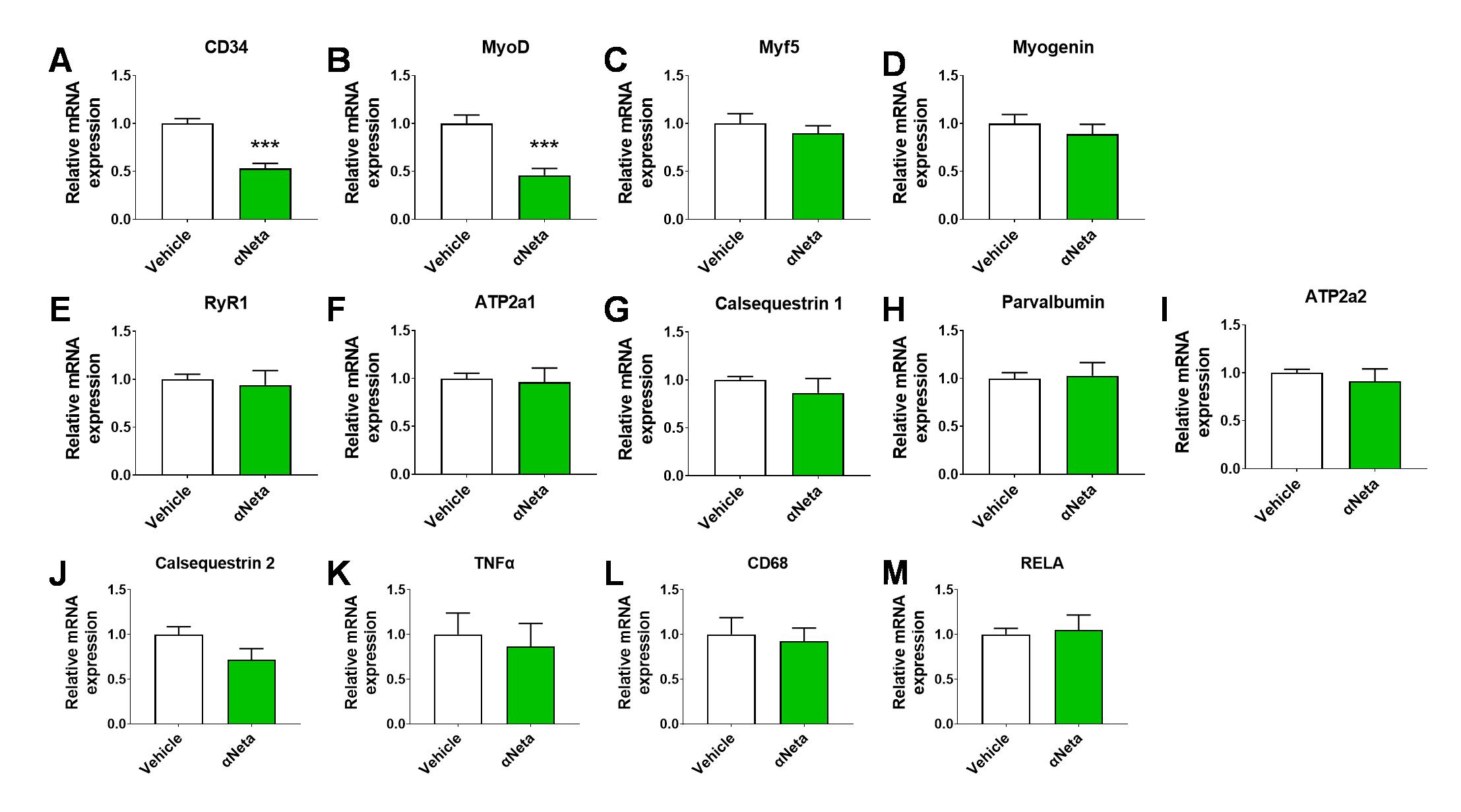

Supplement: Supplementary file 3 [file Image2.JPEG]

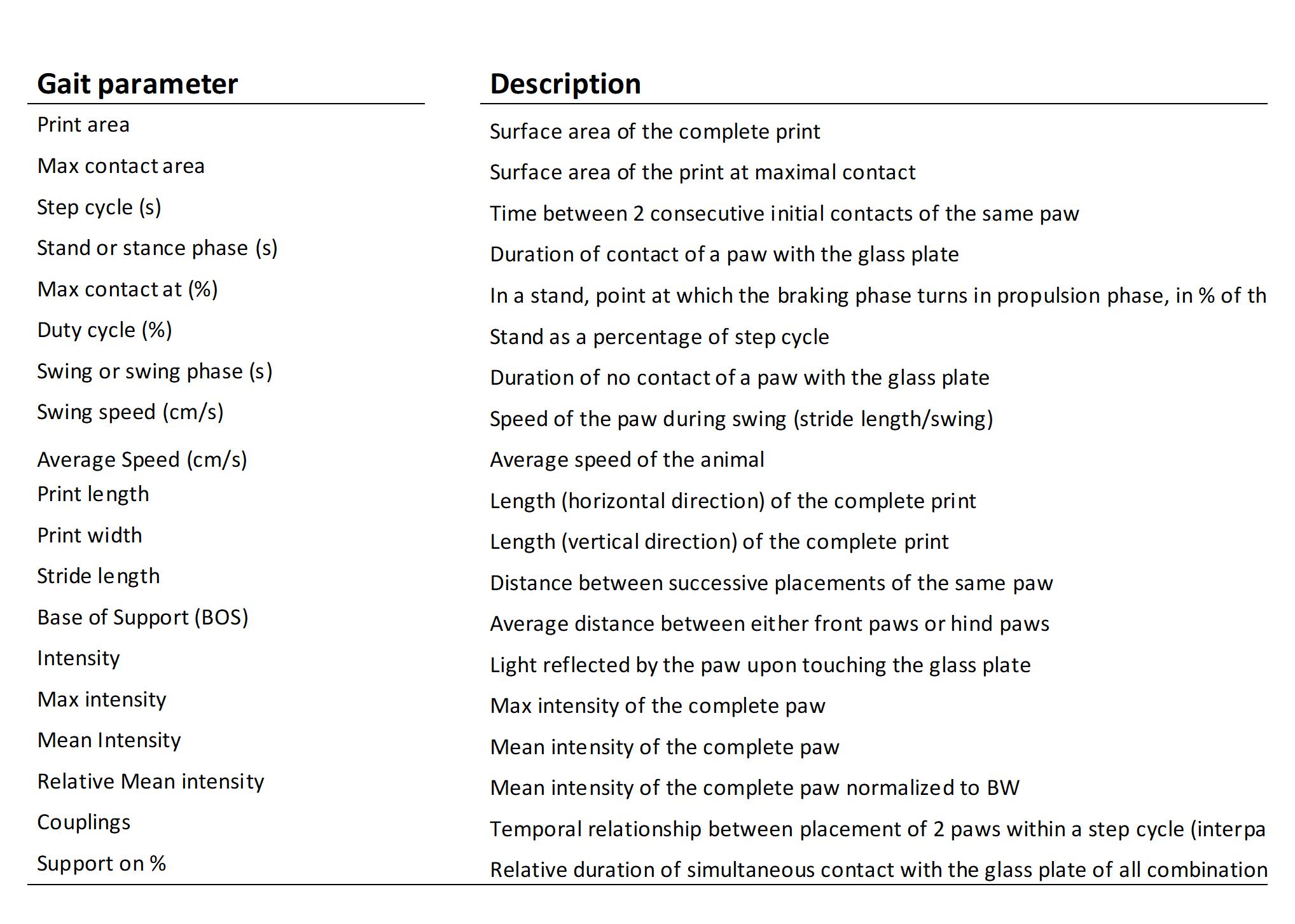

Supplement: Supplementary file 4 [file Image4.jpg]
